# Evaluation of Selenium Levels and Mean Platelet Volume in Patients with Simple Febrile Convulsion

**Published:** 2014-07-02

**Authors:** Mahmut Abuhandan, Abdullah Solmaz, Suleyman Geter, Cemil Kaya, Bulent Guzel, Ilhan Yetkin, Bulent Koca

**Affiliations:** Department of Pediatrics, Medical Faculty, Harran University, Sanliurfa, Turkey

**Keywords:** Febrile Convulsion, Selenium, Platelet: Mean Platelet Volume, Antioxidant

## Abstract

***Objective:*** This study aimed to evaluate serum selenium levels and mean platelet volume in children who experience simple febrile convulsion.

***Methods:*** The study comprised 42 patients diagnosed with simple febrile convulsions and a control group of 30 healthy children. Blood samples were taken following a febrile convulsion. Selenium levels in the serum of both the patients and control subjects were measured with the hydride formation method on an atomic absorption spectrometry device and mean platelet volume was evaluated.

***Findings:*** When the mean values of the febrile convulsion patients were compared with those of the control group, the mean selenium levels and thrombocyte count were found to be statistically significantly low (*P*=0.002, *P*=0.01 respectively) and the mean platelet volume values were statistically significantly high (*P*=0.002).

***Conclusion:*** While low serum selenium levels cause the onset of a febrile seizure in patients with simple febrile convulsion, it is thought that the increased mean platelet volume shows infection activity causing febrile convulsion.

## Introduction

Febrile convulsion (FC) is defined as a type of seizure accompanied by fever which is seen in children aged 6 months to 5 years with no history of electrolyte imbalance and infection in central nervous system^[^^1^^]^. Although seizures are generally benign in character, care and attention and is appropriate, as occasionally seizures may have properties of recurrence and as there is a risk of the seizures becoming epileptic^[^^1^^]^. The pathogenesis of febrile convulsion is not as yet fully understood. Attempts have been made to explain why fever experienced by all children, develops into convulsion in only some of them. Studies have been conducted on children with febrile convulsion related to interferon-alpha^[^^2^^]^, prolactin^[^^3^^]^, low levels of cortisol^[^^3^^]^, anemia^[^^4^^]^ and zinc deficiency^[^^5^^]^, but the role of all of these in the pathogenesis of FC remains controversial. 

 It has been suggested that oxidative damage in neurological diseases and changes in the antioxidant defense system which result in increased lipid peroxidation may play a role in the pathogenesis of epilepsy and febrile convulsion^[^^6^^]^. Selenium, an important antioxidant for humans, has been studied in childhood epilepsy and particularly in resistant epilepsy and its levels were found to be low compared to those of normal children. Thus, it has been proposed that selenium may become a part of epilepsy treatment in future^[^^7^^]^.

 Platelets play a fundamental role in thrombosis and homeostasis. However, recent studies have revealed that platelets play an even greater role in infection and inflammation^[^^8^^]^. Activated platelets cause an increase in the size of platelets by expressing inflammatory factors such as chemokines and cytokines. In other words, increased mean platelet volume is an indicator that thrombocytes have been activated^[^^9^^]^. Thrombocyte size measured as mean platelet volume (MPV), thrombocyte aggregation, thromboxane A2, thrombocyte factor 4 and thromboglobulin expression are good indicators of specific activities of thrombocytes^[^^9^^]^. The changes in MPV have been studied in several diseases^[^^10^^,^^11^^]^.

 This study aimed to evaluate the changes of the mean platelet volume values and serum selenium levels which follow a convulsion in patients with simple febrile convulsion.

## Subjects and Methods

The study comprised 42 patients aged between 9 months and 60 months who presented at the pediatric clinic and the pediatric emergency clinic between 1 January 2012 and 1 April 2013 having undergone a generalized tonic clonic convulsion lasting less than 15 minutes and not recurring within 24 hours and who were then diagnosed with simple febrile convulsion. The control group comprised 30 healthy children aged 8 months to 60 months who were brought to the general pediatrics clinic for vaccination and/or a routine health check and who had no history of convulsions, epilepsy or neurological impairment. Informed consent was obtained from the parents of all the participants. Approval for the study was granted by the Local Ethics Committee. After taking a detailed anamnesis, the health status of all the children was determined from physical examination.

 Those with metabolic disease, hematologic disorders, chronic disease, a history of asphyxia and afebrile convulsion, neurological sequelae, neurological findings following a convulsion and those with a diagnosis of degenerative central nervous system and demyelinisation disease were excluded from the study.

 Blood samples were taken 2 hours after the febrile convulsion. For complete blood count an automatic blood count device (Abbott Celldyn 3500 Ill, USA) was employed in all febrile convulsion patients and all healthy control group children. The blood samples were centrifuged at 3500 rpm for 10 minutes, and then the formed elements were discarded with the tube. Part of the serum samples was stored at -80ºC. The remaining serum samples were tested for electrolytes, kidney and liver function (Abbott Aeroset, Abbott Diagnostics, Abbott Park, IL, USA) on the same day and on the study day, serum selenium levels were measured with the hidrure generation method on an atomic absorption spectometry device from the serum samples stored at -80ºC.

 Data were analyzed using SPSS (Statistical Package for the Social Sciences, version 11.5 for Windows, SPSS® Inc, Chicago, IL). Distribution of parametric variables was assessed with one-sample Kolmogorov–Smirnov test and all parametric variables were found to be normally distributed. The results were presented as mean±standard deviation. Demographic data was performed using chi-square test. Independent samples t test was used. Binary logistic regression analysis was performed to find independent predictors of patients with simple febrile convulsions. Receiver operating curve (ROC) analysis was performed to assess the value of selenium levels to detect the cutoff value of the risk of febrile convulsion.  A two-tailed *P* value of less than 0.05 was considered statistically significant.

## Findings

The 42 patients with simple febrile convulsion included in the study consisted of 30 (71.4%) males and 12 (28.6%) females with a mean age of 2.6±1.2 years. The healthy control group comprised 16 (53.3%) males and 14 (46.7%) females with a mean age of2.4±1.2 years. No statistically significant difference was found between the two groups in terms of age or gender (*P*>0.05) (Table 1).

**Table 1:** Selenium, MPV, platelet count and demographic data of the patient group and the control group

**Variable**	**Patient group (n=42)** **Mean (SD)**	**Control group (n=30)** **Mean (SD)**	***P. *** **value**
**Gender, (Male/Female)**	30/12	16/14	0.09
**Age (years)**	2.7 (1.5)	2.5 (1.2)	0.5
**Selenyum (** **μg/L** **)**	44.1 (13.4)	53.5 (10.9)	0.002
**MPV (/fL)**	6.4 (1.1)	5.8 (0.5)	0.002
**Platelet (/μL)**	353.4 (105.3)	407.2 (69.4)	0.01

Student t test for Independent Samples was used. SD: Standard deviation; MPV: mean platelet volume

Both viral and bacterial infection findings of upper respiratory tract (tonsillitis, otitis) existed in all patients. 

 When the mean selenium levels of the febrile convulsion patients were compared with those of the control group, they were found to be statistically significantly lower (*P*=0.002) (Table 1).

 While the MPV values were found to be statistically significantly higher compared to those of the control group (*P*=0.002), total platelets counts were found to be significantly lower (*P*<0.01) (Table 1).

**Fig. 1:**
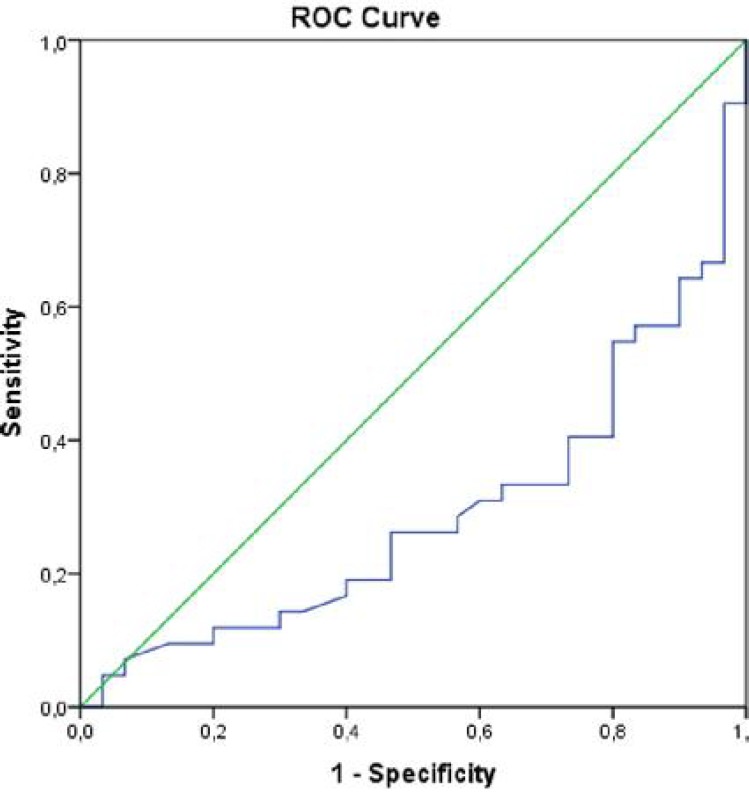
Graph demonstrating receiver operating curve (ROC) analysis of selenium levels with area under the curve of 0.290

 Binary logistic regression analysis revealed that decreased selenium level was an independent factor (B=-0.063, SE=0.022, Wald=7.99, *P*=0.005). In ROC-curve analysis, selenium levels below 49.05 mg/ml showed 73.3% sensitivity and 66.7% specificity for the risk of developing simple febrile seizure [area under the curve of 0.290 (95% Confidence Interval: 0.17-0.41)] (Fig. 1).

## Discussion

To the best of our knowledge, the present study is the first in literature to investigate the relationship between Selenium, MPV, platelets and febrile seizures; and gave intriguing results: (i) Se and MPV values were found to be lower, (ii) platelet counts were found to be higher than control group, (iii) decreased selenium levels were independent predictors for simple febrile convulsions, and (iv) analysis of ROC for sensitivity and specificity revealed that the selenium levels below 49.05 mg/ml showed 73.3% sensitivity and 66.7% specificity for the risk of developing simple febrile seizure.

 Although febrile convulsion is one of the most frequently occurring forms of convulsion seen in childhood, the pathogenesis is not yet fully understood. In recent years, studies have been conducted on the oxidative damage, changes in antioxidant enzymes and lipid peroxidation in neurological diseases which accompany epilepsy and convulsions.

There are data suggesting that the occurrence of active oxygen metabolites and decreased activity of the antioxidative defense mechanisms can increase the risk of seizures^[^^12^^,^^13^^]^. It is thought that oxidative stress may play a role as a mechanism in the etiology of neuronal generation which is triggered by convulsion^[^^14^^]^.

 In previous studies it has been understood that there exists a cause and effect relationship between oxidative stress, reactive oxygen species production and epileptic seizures. Due to the protective effect against oxidative damage and the life-extending effect on neuronal cells of selenium and selenoproteins, when the serum selenium levels of epileptic and healthy children have been compared, those of children having suffered seizures have been found to be lower^[^^7^^]^. In studies which have compared mean selenium levels of febrile convulsion patients with those of a healthy control group, the mean selenium levels of febrile convulsion patients have been reported to be significantly low^[^^15^^]^. In our study, the mean serum selenium levels of the febrile convulsion patients were found to be significantly lower compared to those of the control group. Therefore, it can be said that free radicals are both the cause and result of epileptic seizures, as the serum selenium level may fall associated with the use of selenium as an antioxidant to neutralize oxidative damage which has been created as a result of oxygen radicals produced in the nervous system^[^^7^^]^ just as in mitochondria which occur all over the body from oxidative phosphorylation. 

 In events of tissue oxygenation and nutritional impairment and in cases such as diabetes mellitus^[^^16^^]^, myocardial infarct^[^^17^^]^, ischemic shock^[^^18^^]^, cigarette smoking^[^^19^^]^, renal artery stenosis^[^^20^^]^ and hypertrophic dilated cardiomyopathy^[^^21^^]^, thrombocyte volume has been reported to increase. MPV values have been reported to fall in diseases such as inflammatory bowel disease^[^^22^^]^, pneumonia^[^^11^^]^, Kawasaki disease ^[^^23^^]^ and acute phase Familial Mediterranean Fever ^[^^10^^]^. In a study by Ozaydin et al MPV values of simple febrile convulsion patients were found to be higher than those of complex febrile convulsion patients^[^^24^^]^. 

 In the current study, the MPV values of the febrile convulsion patients were compared with the MPV values of the control group and the MPV values of the febrile convulsion patients were found to be significantly higher than those of the control group. The mean thrombocyte count was significantly lower compared to that of the control group. This may be caused by a reduced number of thrombocytes in circulation resulting from thrombocytes of smaller diameter having been used first. To compensate for the decreased number of platelets, the bone marrow rapidly produces thrombocytes. The mean size of these new thrombocytes is greater and this may be a reason for the increase in MPV. Thrombocyte volume is a parameter for defining thrombocyte function and thrombocytes of greater volume are hemostatically active.

 In thrombocytopenic patients with associated thrombocyte consumption or thrombocyte loss, higher MPV values are seen and this increase is thought to arise from thrombocyte consumption ^[^^25^^]^. Another reason, is that thrombocytes as immune cells like polymorph nuclear leukocytes, play a significant role in the phagocytosis and chemotaxis of micro-organisms such as viruses, bacteria and parasites and by expression of some inflammatory cytokines, these functions have been shown to be restored^[^^26^^]^. The increased size of platelets may be due to activated platelets expressing inflammatory factors such as chemokines and cytokines^[^^27^^]^. As thrombocyte function and volume are correlated, they contain more granules and in metabolic and enzymatic terms are more active^[^^28^^]^, so MPV may be a simple inflammatory marker for inflammation^[^^29^^,^^30^^]^.

## Conclusion

While serum selenium levels and thrombocyte count were found to be statistically significantly low in FC cases compared to the control group, the MPV values were found to be high. It is thought that there may be a reduction in selenium associated with its consumption as an antioxidant to neutralize the increased oxidative damage in febrile convulsions. It can be said that this drop in selenium level causes the onset of a seizure. In the event of acute inflammation and infection which create an immune response, increased MPV, which is associated with structural changes in thrombocytes, even showing an anti-microbial effect, shows infection activity causing simple febrile convulsions.
